# Familiale Belastungsfaktoren für die emotionale Entwicklung junger Kinder

**DOI:** 10.1007/s00103-023-03730-3

**Published:** 2023-07-04

**Authors:** Sabine Walper, Susanne M. Ulrich, Heinz Kindler

**Affiliations:** Deutsches Jugendinstitut, Nockherstr. 2, 81541 München, Deutschland

**Keywords:** Adverse Childhood Experiences, Armut, Elternstress, Kindliches Wohlbefinden, Frühe Kindheit, Adverse childhood experiences, Poverty, Parenting stress, Child well-being, Early childhood

## Abstract

**Hintergrund:**

Belastende Kindheitserfahrungen (Adverse Childhood Experiences – ACE) sind Risikofaktoren für die körperliche und psychische Gesundheit, deren Folgen bis ins Erwachsenenalter reichen können. Basierend auf Befunden zu den Effekten ausgewählter ACE sowie deren Kumulation wird hier untersucht, welche Effekte unterschiedliche familiale Belastungslagen auf die Ausprägung negativer Emotionalität von Kindern im Säuglings- und Kleinkindalter haben.

**Methoden:**

Datenbasis ist die repräsentative Elternbefragung „Kinder in Deutschland 0–3 – KiD 0–3“ (*N* = 5583) mit Folgebefragung einer Teilstichprobe nach 2 Jahren (*n* = 681). Anhand von 14 Belastungsfaktoren werden unbelastete, sozioökonomisch belastete, elternstressbelastete und mehrfach belastete Familien unterschieden.

**Ergebnisse:**

Kinder in mehrfach belasteten Familien haben das höchste Risiko hoher negativer Emotionalität (im Vergleich zu unbelasteten Familien: Odds Ratios [OR] zwischen 13,00 und 6,81), unter Kontrolle demografischer Merkmale, kindbezogener Belastungsfaktoren (z. B. exzessives Schreien) und Kindheitsbelastungen der Bezugsperson. Auch in elternstressbelasteten Familien ist das Risiko negativer Emotionalität der Kinder signifikant erhöht (OR zwischen 8,31 und 6,95), während Kinder aus primär sozioökonomisch belasteten Familien (ohne Konflikte und Elternstress) keine erhöhte negative Emotionalität aufwiesen. Längsschnittanalysen der wiederbefragten Teilstichprobe zeigen, dass Veränderungen der Anzahl von Belastungsfaktoren mit Veränderungen kindlicher negativer Emotionalität einhergehen.

**Diskussion:**

Die Befunde zu ACE aus der internationalen Forschung bestätigen sich damit für den deutschsprachigen Raum und speziell für die frühe Kindheit. Dies unterstreicht die Bedeutung eines gut ausgebauten Systems Früher Hilfen.

## Abstract (en)

**Background:**

Adverse childhood experiences (ACE) have been pointed out as risk factors for physical and mental health, with consequences extending from childhood into adulthood. Based on research regarding the effects of selected ACE as well as the accumulation of ACE, this article investigates how different types of family stressors are linked to children’s negative emotionality in infancy and early childhood.

**Methods:**

Data are from the KiD 0–3 study (*N* = 5583) and the follow-up of a subsample after 2 years (*n* = 681). Based on 14 stress factors, we distinguish families with no/little stressors, socioeconomic stressors, parenting stressors, and multiple stressors.

**Results:**

Children in multiply stressed families have the highest risk of high negative emotionality (compared to unstressed families: Odds Ratios [OR] ranging from 13.00 to 6.81), controlling for demographic characteristics, child-related stress factors (e.g., excessive crying), and caregiver childhood stress. Children in families primarily characterized by parenting stress also showed a significantly increased risk of high negative emotionality (OR ranging from 8.31 to 6.95), whereas this did not hold for children from socioeconomically stressed families (without parenting stress) compared to those from unstressed families. Longitudinal analyses of the follow-up subsample showed that changes in the number of stressors were also associated with parallel changes in children’s negative emotionality.

**Discussion:**

These results confirm findings from international research on ACE in Germany and for early childhood. They underline the importance of a well-developed early intervention system.

## Hintergrund

Kinder wachsen unter sehr unterschiedlichen Lebensbedingungen auf und bringen ihrerseits auch sehr unterschiedliche individuelle Voraussetzungen für den Umgang mit den jeweiligen äußeren Gegebenheiten mit. Geringe sozioökonomische Ressourcen der Familie, eine konflikthafte Trennung der Eltern [1–3, 5–7], mangelnde Zuwendung und Anregung seitens der Eltern gehören zu den Faktoren, die das Aufwachsen von Kindern überschatten können [1–3]. Gleichzeitig verfügen Kinder in unterschiedlichem Maß über Kompetenzen und Ressourcen, die es ihnen erleichtern, mit schwierigen Bedingungen zurechtzukommen [4]. Und nicht nur Erwachsene, sondern auch Kinder haben eine durch ihr Temperament und ihre Persönlichkeit gefärbte „Brille“, durch die sie ihre Umwelt auf je eigene Weise erleben [5]. Vor diesem Hintergrund ist die Frage nach dem, was „glückliche“ und „unglückliche“ Kindheiten ausmacht, nicht einfach zu beantworten, denn in ihr schwingt nicht nur der Blick auf die Kontexte des Aufwachsens, sondern auch der Blick auf das subjektive Erleben mit, das nur in begrenztem Maße die äußeren Gegebenheiten reflektiert.

Der Großteil der Forschung hat sich bislang weniger mit der positiven Seite glücklicher Kindheiten befasst, obwohl im Zuge der Glücksforschung und der Forschung zu kindlichem Wohlbefinden (z. B. [6]) die positiven Seiten der Entwicklungsbedingungen von Kindern stärker in den Vordergrund gerückt wurden. Wesentlich mehr Aufmerksamkeit galt belastenden Bedingungen des Aufwachsens und resultierenden Beeinträchtigungen der Entwicklung und psychischen Gesundheit von Kindern. Der vorliegende Beitrag gibt zunächst einen Überblick über relevante psychosoziale Belastungen und ergänzt diese durch Befunde einer repräsentativen deutschen Studie (Kinder in Deutschland – KiD 0–3) zur Entwicklung negativer Emotionalität von Kleinkindern in unterschiedlichen Risikolagen. Negative Emotionalität mit Symptomen wie häufigen Wutanfällen, trotzigem Verhalten und schwerer Beruhigbarkeit sowie geringen Anzeichen positiver Emotionen wie Zufriedenheit und Freude indiziert kindliche Probleme in der Emotionsregulation, die einem schwierigen Temperament zugeordnet werden, aber auch als Vorläufer für Beeinträchtigungen der psychischen Gesundheit zu sehen sind (s. unten).

### Ausgewählte Risikofaktoren

#### Mangelnde sozioökonomische Ressourcen

Sozioökonomische Faktoren haben deutlichen Einfluss auf die Handlungsspielräume von Familien, deren Alltagsgestaltung und das Wohlergehen von Eltern und Kindern. Geringe sozioökonomische Ressourcen, festgemacht an niedriger formaler Bildung der Eltern, Einkommensarmut oder einem insgesamt geringen sozioökonomischen Status, gehen oft mit vermehrten Sorgen, Ängsten und psychischen Problemen der Eltern [7], Beeinträchtigungen des Familien- und Erziehungsklimas [8–10] und vielfältigen Nachteilen für die Kinder einher. Das zeigt sich etwa in deren früher Kompetenzentwicklung, ihrem Bildungsverlauf, Wohlbefinden und ihrer Gesundheit [2, 11, 12].

Neben Beeinträchtigungen der allgemeinen Gesundheit finden sich bei niedrigen sozioökonomischen Ressourcen vor allem Nachteile hinsichtlich der psychischen Gesundheit von Kindern und Jugendlichen [13] wie auch ihres Temperaments [14], das für die psychische Gesundheit relevant ist [15]. Dass finanziellen Einschränkungen hierbei eine ursächliche Rolle zukommt, legen u. a. Längsschnittdaten nahe, nach denen mit dem Beginn finanzieller Belastungen auch Beeinträchtigungen des Wohlbefindens und der psychischen Gesundheit zunehmen [16, 17]. Auch Interventionsstudien zeigen, dass der Einkommenssituation für die Entwicklung der Kinder und das Wohlergehen der Familien besondere Bedeutung zukommt [18].

Wie das Family Stress Model (FSM; [8, 19]) herausstellt, spielen familiäre Reaktionen auf sozioökonomische Belastungen – und speziell der finanzielle Stress sowie die hieraus resultierenden Belastungen der Beziehungen und Interaktionen – eine zentrale Rolle für Beeinträchtigungen der Gesundheit und der Entwicklung der Kinder aus armutsbetroffenen Familien. Finanziell bedingter Stress und emotionale Belastungen der Eltern unter ökonomischem Druck können deren Aufmerksamkeit gegenüber kindlichen Bedürfnissen und unterstützendes Erziehungsverhalten einschränken, während harsches Erziehungsverhalten wahrscheinlicher wird [20], insbesondere wenn soziale Ressourcen der Eltern fehlen [21]. Vor allem vermittelt über solche Belastungen der Beziehungen und Interaktionen, trägt Armut zu Beeinträchtigungen des Wohlergehens von Kindern und Jugendlichen bei (z. B. [9, 22]).

#### Konflikte und Trennung der Eltern

Am Ende des Grundschulalters hat in Westdeutschland jedes vierte und in Ostdeutschland mehr als jedes dritte erstgeborene Kind getrennte Eltern; für rund ein Drittel dieser Kinder galt dies schon bei Geburt [23]. Obwohl Trennungen bzw. Scheidungen kaum noch stigmatisiert sind und im Einzelfall auch positive Entwicklungsmöglichkeiten eröffnen können, überwiegen im statistischen Durchschnitt eher die Risiken für das Wohlergehen und eine positive Entwicklung der betroffenen Kinder [3, 24]. Einige Befunde deuten auf eine besondere Vulnerabilität der sozioemotionalen Entwicklung in der frühen Kindheit gegenüber elterlichen Trennungen und ihren Begleitumständen hin [25]. Auch neue Partnerschaften der Eltern können zusätzliche Anpassungen der Kinder erforderlich machen und belastend sein [26].

Mit einem vierfach erhöhten Armutsrisiko Alleinerziehender im Vergleich zu Paarfamilien fallen die ökonomischen Nachteile Alleinerziehender in Deutschland stärker aus als in den meisten anderen europäischen Ländern [23]. Aber nicht nur die vielfach ungünstige finanzielle Situation Alleinerziehender, sondern auch anhaltende Probleme zwischen den Eltern erweisen sich als wichtige Belastungsmomente für Kinder. Das Miterleben von Konflikten und eine zeitweise verringerte elterliche Fürsorge- und Erziehungsfähigkeit der Eltern sind wichtige Vermittlungsmechanismen für negative Auswirkungen familiärer Instabilität [27, 28]. Hierbei sind Kinder, die intensivere oder chronifizierte Elternkonflikte nach einer Trennung miterleben, im Mittel stärker belastet als Kinder, bei denen dies nicht der Fall ist [29]. Allerdings belasten chronische Konflikte, insbesondere in der Zusammenarbeit der Eltern bei der Fürsorge und Erziehung, die Kinder auch dann, wenn es nicht zu einer Trennung der Eltern kommt (z. B. [29]).

#### Gewalt und Vernachlässigung

Von familiärer Gewalt und Vernachlässigung in mindestens einer Form (körperliche, psychische oder sexuelle Gewalt, Vernachlässigung) sind laut einer bundesweit repräsentativen Erhebung immerhin 13 % der Kinder und Jugendlichen betroffen [30]. Zwar finden sich sozioökonomische Risiken oder Elternkonflikte häufiger, aber das Schädigungspotenzial von familiärer Gewalt und Vernachlässigung ist besonders hoch: Der Anteil psychischer Erkrankungen in der Bevölkerung, der hierauf zurückzuführen ist („population attributable fraction“) beträgt in internationalen Studien über 20 % (z. B. [31]).

Zu den familiär Betroffenen kommen noch Kinder und Jugendliche hinzu, die erhebliche Gewalt und Ausgrenzung durch Gleichaltrige, sexuelle Gewalt durch Erwachsene außerhalb der Familie oder schwere Gewalt in ersten Partnerschaften erleben. Die psychischen Folgewirkungen solcher Erfahrungen wurden in Längsschnittstudien wiederholt belegt (z. B. [32, 33]). Für alle Formen von Gewalt und Vernachlässigung zeigt sich – wenngleich in unterschiedlichem Ausmaß – ein sozialer Gradient. Das bedeutet, dass Kinder aus sozioökonomisch benachteiligten Familien mehr Gewalt und Vernachlässigung erfahren als Kinder aus besser gestellten Familien [34]. Zudem erleben Kinder, deren Eltern sich trennen oder erhebliche Konflikte miteinander haben, überdurchschnittlich häufiger Misshandlung bzw. Vernachlässigung [35]. Signifikante Schädigungseffekte von Gewalt oder Vernachlässigung bleiben aber auch dann bestehen, wenn für sozioökonomische Faktoren und Familienformen kontrolliert wird (z. B. [36]).

Neben gesundheitlichen Auswirkungen von Gewalt und Vernachlässigung [37] finden sich durchgängig, auch in deutschen Studien, negative Zusammenhänge zur Lebenszufriedenheit und Lebensqualität (z. B. [38]). Längsschnittstudien zeigen die stärksten Belastungseffekte für früh einsetzende, chronische Gewalt und Vernachlässigung auf (z. B. [39]). Ungünstige Temperamentsfaktoren wie negative Emotionalität werden durch Erfahrungen von Gewalt und Vernachlässigung verstärkt und erschweren zusätzlich deren Bewältigung [40].

#### Die Kumulation von Belastungslagen

Einzelne Belastungsfaktoren treten selten isoliert auf und vielfach sind bei einem Zusammentreffen mehrerer solcher Faktoren auch stärkere Beeinträchtigungen der Entwicklung und Gesundheit von Kindern zu verzeichnen. Dem trägt vor allem das Konzept der *Adverse Childhood Experiences* (ACE) Rechnung, das den Fokus insbesondere auf die Kumulation von Belastungsfaktoren richtet und deren Relevanz für Beeinträchtigungen der körperlichen und psychischen Gesundheit aufzeigt (z. B. [41, 42]). Typischerweise einbezogen werden Faktoren wie Armut, elterliche Konflikte und Trennungen, psychiatrische Störungen, Substanzmissbrauch, gerichtliche Verurteilungen/Gefängnisaufenthalte eines Elternteils, aber auch Kindesmisshandlung und -missbrauch.

In der Vergangenheit war der Fokus zumeist auf die Folgen retrospektiv erfasster ACE für die Gesundheit im Erwachsenenalter gerichtet (z. B. [43, 44]), aber international sind zunehmend auch Langzeitstudien verfügbar, die es erlauben, Effekte von Kindheitserfahrungen bis ins Erwachsenenalter zu verfolgen (z. B. [45]). Unter Einbezug beider Studienarten erbrachte eine Metaanalyse, dass bei einer Kumulation von 4 ACE signifikante gesundheitliche Belastungen in allen Bereichen zu verzeichnen sind, mit starken Effekten im Bereich der psychischen Gesundheit und des problematischen Alkoholgebrauchs (OR über 3 bis 6) und stärksten Effekten für problematischen Drogengebrauch und Gewalt (OR über 7; [41]). Entsprechende Dosiseffekte mit stärkeren Effekten bei höherer Anzahl von ACE zeigen sich ebenso bei Kindern und Jugendlichen [46], sowohl hinsichtlich internalisierenden und externalisierenden Problemverhaltens sowie Diagnosen der Aufmerksamkeitsdefizit‑/Hyperaktivitätsstörung (ADHS) in der mittleren Kindheit [42] als auch hinsichtlich chronischer Erkrankungen und geringer Bildungsteilhabe [47]. Analog hierzu lassen sich Befunde zu Beeinträchtigungen der psychischen Gesundheit von Kindern und Jugendlichen bei einer Kumulation elterlicher Belastungsfaktoren interpretieren [48], da diese auch den familiären Entwicklungskontext der Kinder beschreiben. Insgesamt ist die Bedeutung von ACE für eine lebenslang verschlechterte körperliche und psychische Gesundheit gut dokumentiert.

In einer Reihe von Arbeiten wurden auch spezifische Konstellationen von Belastungsfaktoren untersucht [49, 50]. Witt et al. (2019) ermittelten anhand latenter Klassenanalyse 4 Muster von ACE: keine/minimale ACE (77,1 %), familiäre Probleme (10,7 %), Kindesmisshandlung (7,4 %) und multiple ACE (4,8 %). In dieser Reihenfolge der Gruppen stieg der Anteil derer mit geringer Lebensqualität und Ängsten, Depressivität und/oder körperlicher Aggression mit jeweils höchsten Werten in der Gruppe mit multiplen ACE [30, 51]. In der britischen längsschnittlichen ALSPAC-Studie (Avon Longitudinal Study of Parents and Children; [49]) ergaben sich mittels latenter Klassenanalyse 5 Typen: geringe ACE (55 %), elterliche Trennung und psychische Probleme der Mutter (18 %), psychische Probleme der Eltern, Verurteilungen und Trennung (15 %), Misshandlung und psychische Probleme (6 %) und Poly-Probleme (6 %). Wie bei Witt et al. war Armut hierbei nicht einbezogen. Armut während der Schwangerschaft erwies sich jedoch als besonders eng mit dem Cluster Poly-Probleme verbunden (OR 9,15) und war auch bei den anderen ACE-Clustern 2‑ bis 4fach häufiger gegeben als bei geringen ACE.

Die 5 Belastungstypen bieten allerdings keine trennscharfe Zuordnung der Risikofaktoren und sind durch den fehlenden Einbezug sozioökonomischer Faktoren nur eingeschränkt aussagekräftig. Deutlich klarere Belastungsprofile ergaben sich in Analysen der umfangreichen Repräsentativbefragung von Eltern junger Kinder „Kinder in Deutschland – KiD 0–3“ [50], wobei auch das Armutsrisiko und familienstrukturelle Faktoren einbezogen waren. Latente Klassenanalysen von 25 Belastungsfaktoren, die sich in Längsschnittstudien als prädiktiv für Entwicklungsprobleme und Kindesmisshandlung erwiesen haben [52], erbrachten 4 Typen von Belastungsmustern: eher unbelastete Familien (58,9 %), sozioökonomisch belastete Familien (18,8 %), elternstress- und konfliktbelastete Familien (17,2 %) und mehrfach belastete Familien, die u. a. sowohl sozioökonomische Risiken als auch vermehrten Elternstress und Konflikte aufwiesen (5,2 %). Da der Fokus auf Belastungen der Eltern bezogen war, gingen hierbei auch Besonderheiten der Kinder wie Erkrankung oder Behinderung, problematisches Schreiverhalten oder negative Emotionalität ein, statt – wie im Folgenden – nach emotionalen Belastungen der Kinder in Abhängigkeit vom Belastungsprofil der Familie zu fragen.

### Belastungsprofile in KiD 0–3 nach Lorenz et al. (2020; [50])

In der bundesweit repräsentativen Elternbefragung KiD 0–3 wurden 25 Belastungsfaktoren herangezogen, um unterschiedliche Belastungsprofile von Eltern junger Kinder in Deutschland zu ermitteln. Ausgewählt wurden Belastungsfaktoren, die sich in Längsschnittstudien als valide Prädiktoren für Erziehungsschwierigkeiten, Entwicklungsauffälligkeiten oder Kindeswohlgefährdung erwiesen haben. Die Belastungsfaktoren charakterisieren folgende Aspekte:

- die *Lebenssituation der Familie* (niedrige Bildung, Armutsrisiko, beengte Wohnverhältnisse, alleinerziehend/Stieffamilie, Partnerschaftskonflikte/‑gewalt, fehlende soziale Unterstützung, mehrere kleine Kinder),
- die *persönlichen Voraussetzungen für die Bewältigung der Fürsorgeanforderung* (Zweifel an der eigenen erzieherischen Kompetenz, negative Kindheitserfahrungen, junges Alter der Mutter von höchstens 21 Jahren, Anzeige wegen eines Gewaltdelikts),
- die *psychische Gesundheit des befragten Elternteils* (Impulsivität/Ärgerneigung, Depression oder Angst, Hinweise auf Substanzmissbrauch),
- *besondere Fürsorgeanforderungen durch das Kind* (perinatale Probleme, negative Emotionalität, problematisches Schreiverhalten, Behinderung oder Erkrankung),
- *problematisches Fürsorgeverhalten* (Schwierigkeiten, sich in das Kind einzufühlen, Tendenz zur Überreaktion, körperliche Misshandlung, Vernachlässigung).

Mittels einer latenten Klassenanalyse konnten anhand statistischer Informationskriterien 4 Gruppen mit unterschiedlichen Belastungskonstellationen identifiziert werden. Über die Wahrscheinlichkeiten, mit denen die einzelnen Belastungen in den unterschiedlichen Gruppen auftreten, können die Gruppen wie folgt inhaltlich beschrieben werden:

- *eher unbelastete Familien:* kaum Belastungsmerkmale,
- *sozioökonomisch belastete Familien:* z. B. Armutsrisiko, Leben in beengten Wohnverhältnissen oder niedrige Bildung,
- *Eltern mit erhöhtem elterlichen Stress und Konfliktbelastung:* z. B. Zweifel an der elterlichen Kompetenz, vermehrte Schwierigkeiten, sich in das Kind einzufühlen, Ärgerneigung sowie Partnerschaftskonflikte und -gewalt,
- *hochbelastete Familien:* sowohl sozioökonomische Belastungen als auch Elternstress und Konflikte.

Die Wahrscheinlichkeit, dass Familien Sozialhilfebezug berichten, beträgt in der Gruppe der eher unbelasteten Familien nur 2,99 %, in der Gruppe der elternstress- und konfliktbelasteten Familien 6,74 %, in der Gruppe der sozioökonomisch belasteten Familien 66,02 % und in der Gruppe der hochbelasteten Familien 85,95 %.

### Fragestellung

Anknüpfend an die oben genannten Befunde greifen wir die Frage nach unterschiedlichen Belastungstypen und deren Bedeutung für das psychische Wohlbefinden von jungen Kindern im Alter von bis zu 3 Jahren auf. Für dieses sehr junge Alter fehlen bislang noch entsprechende Befunde. Hierbei fokussieren wir die negative Emotionalität von Kindern, die als zentraler Aspekt psychischer Gesundheit im Kleinkindalter herausgestellt wurde [53]. Ausgehend von Belastungsgruppen analog zur o. g. Typisierung von Lorenz et al. [50], aber ohne Einschluss von Merkmalen der Kinder, vergleichen wir 4 unterschiedliche Belastungsgruppen der Familien hinsichtlich der negativen Emotionalität von Kindern. Dieser Vergleich erfolgt sowohl querschnittlich anhand der bundesweit repräsentativen Stichprobe von Kindern im Alter bis zu 48 Monaten der Studie „Kinder in Deutschland – KiD 0–3“ als auch längsschnittlich anhand einer reduzierten Teilstichprobe.

Auf Basis der bisherigen Befunde auch zum Family Stress Model, die vor allem die zentrale Bedeutung der Familiendynamik und Eltern-Kind-Beziehung für die Entwicklung und psychische Gesundheit der Kinder herausgestellt haben (z. B. [9, 54]), erwarten wir im Quer- und Längsschnitt stärkere Belastungen von Kindern aus Familien, die elternstress-/konfliktbelastet oder mehrfach belastet sind, als bei Kindern aus unbelasteten oder ausschließlich sozioökonomisch belasteten Familien. Hinsichtlich der Längsschnittanalysen erwarten wir, dass eine Zunahme von familialen Belastungsfaktoren mit einer Zunahme negativer Emotionalität der Kinder einhergeht.

## Methoden

### Stichprobe

Die bundesweit repräsentative Hauptbefragung „Kinder in Deutschland – KiD 0–3“, die 2015 im Rahmen der Begleitforschung zu den Frühen Hilfen vom Nationalen Zentrum Frühe Hilfen (NZFH) erstmals durchgeführt wurde, bildet die Datenbasis der nachfolgenden Analysen. Die Studie KiD 0–3 wurde vom NZFH realisiert. Die Stichprobe wurde zwischen April und September 2015 über 271 Kinderarztpraxen anlässlich der U‑Untersuchungen (U3–U7a) rekrutiert, wobei die Eltern eingeladen wurden, während der Wartezeit einen schriftlichen Fragebogen auszufüllen. Es nahm jeweils mindestens ein leiblicher Elternteil von insgesamt 7549 Kindern im Alter von 0 bis 48 Monaten (M = 14,6 Monate; SD = 12,3; 49,6 % Mädchen) an dieser ersten Erhebung (T1) teil.^1^

Zwei Jahre später wurden alle 2202 Bezugspersonen, die ihre Bereitschaft zu einer Wiederbefragung angegeben hatten, erneut kontaktiert. Den postalisch übermittelten Fragebogen beantwortete jeweils mindestens ein leiblicher Elternteil von insgesamt 909 Kindern (Durchschnittsalter 35,1 Monate, SD = 11,8; 47,3 % Mädchen) in dieser zweiten Befragung (T2).^2^ Während die Ausgangsstichprobe zu T1 ein hohes Maß an Repräsentativität aufweist [55], zeigt die längsschnittliche Stichprobe der Zweitbefragung stärkere Ausfälle bei Familien mit geringen sozioökonomischen Ressourcen und Mehrfachbelastungen (s. unten). So sinkt der Anteil von Familien mit Bezug von Leistungen zur Sicherung des Lebensunterhalts nach dem zweiten Sozialgesetzbuch (SGB II) zu T1 von 19,5 % in der Ausgangsstichprobe auf 11,7 % in der Längsschnittstichprobe und der Anteil von Familien mit Migrationshintergrund von 30,5 % auf 21,7 % (jeweils ungewichtete Daten). Dies wird bei der Interpretation der Befunde zu berücksichtigen sein.

### Definition der Belastungsgruppen

Um die Typisierung von Belastungslagen nach Lorenz et al. [50] zu rekonstruieren, wurden die Kernindikatoren herangezogen, die die Typen der Belastungslagen bestmöglich charakterisieren. Merkmale auf Kindebene wie negative Emotionalität oder problematisches Schreiverhalten wurden ausgeschlossen, da sie als Outcome dienten bzw. damit korrelierten. Als 7 Indikatoren *sozioökonomischer Belastungen *wurden herangezogen: Leistungsbezug nach SGB II, niedrige Bildung, beengte Wohnverhältnisse, alleinerziehend oder mit neuem Partner zusammenlebend, junges Alter der Mutter, ungeplante Schwangerschaft, mehrere Kleinkinder im Haushalt. Trafen mindestens 2 dieser Faktoren zu, so wurde die Familie als „sozioökonomisch belastet“ eingestuft. Als Indikatoren von* Elternstressbelastung* dienten folgende 7 Merkmale: Partnerschaftskonflikte/‑gewalt, innere Wut/Ärger, Depression/Ängste, Zweifel an der erzieherischen Kompetenz, negative Attribution gegenüber dem Kind, Schwierigkeiten, sich in das Kind einzufühlen, Tendenz zur Überreaktion. Trafen mindestens 2 dieser Faktoren zu, so wurde die Familie als elternstressbelastet eingestuft. Anhand der Kombination beider Belastungsmerkmale wurde die Gruppe der *mehrfach belasteten Familien* bestimmt, für die entsprechend mindestens 4 der insgesamt 14 als Kriterien herangezogenen Belastungsmerkmale zutrafen. Elf der Belastungsmerkmale, die in die Typologie von Lorenz et al. eingeflossen sind, blieben hierbei unberücksichtigt, weil sie sich entweder auf die Kinder beziehen, geringe Prävalenz oder geringe Trennschärfe aufwiesen.

*Negative Emotionalität* des Kindes wurde anhand einer Kurzskala erfasst, die ausgehend von der Child Behavior Checklist speziell für das Kleinkindalter entwickelt wurde [53]. Der Indikator umfasst 6 Items (z. B. „Mein Kind ist oft sehr trotzig“) und verfügt über befriedigende interne Konsistenz (*a* = 0,76). Für Analysen zu klinisch relevanter *hoher negativer Emotionalität* wurde die Skala dichotomisiert, wobei ein Summenwert von mindestens 15 Punkten auf einer Skala von 6 bis 23 hohe negative Emotionalität indiziert.

### Statistische Analysen

Für die Gesamtstichprobe wurden querschnittlich zu T1 Effekte der Belastungsgruppen auf hohe negative Emotionalität der Kinder (dichotomer Indikator) geprüft. Hierfür wurden 4 logistische Regressionsanalysen durchgeführt, die jeweils unterschiedliche Prädiktoren einbeziehen. Neben den Belastungsgruppen (Dummy-kodiert) kontrolliert Modell 1 das Alter und Geschlecht des Kindes sowie den Migrationshintergrund der Familie, in Modell 2 und 3 werden mit perinatalen Problemen und einer Behinderung/Erkrankung des Kindes (Modell 2) sowie zusätzlich belastendem Schreiverhalten (Modell 3) kindbezogene Faktoren hinzugenommen und in Modell 4 werden ergänzend unglückliche Kindheitserfahrungen der Bezugsperson berücksichtigt.

Für die Längsschnittanalysen wurde angesichts der insgesamt geringeren Fallzahl und speziell der sehr kleinen Gruppe mehrfach belasteter Familien in der Längsschnittstichprobe auf den Vergleich der Belastungsgruppen verzichtet und stattdessen die Kumulation von Belastungsfaktoren und deren Veränderung betrachtet. Zunächst wurde geprüft, inwieweit sich hohe negative Emotionalität der Kinder zu T2 (dichotomisiert) durch folgende Faktoren vorhersagen lassen: hohe negative Emotionalität der Kinder zu T1, Alter, Geschlecht und Migrationshintergrund der Kinder, die Summe derjenigen Belastungsfaktoren zu T1, die in die Definition der o. g. Belastungsgruppen eingehen, sowie die Veränderung dieser Belastungsfaktoren, soweit sie zu T2 erfasst wurden (Armutsrisiko, geringe Bildung, alleinerziehend/neuer Partner, mehr als 2 junge Kinder, Zweifel an Erziehungskompetenz, geringes Einfühlungsvermögen gegenüber Kind, innere Wut/Ärger, Depression/Ängste, Partnerschaftskonflikte/‑gewalt). Um Veränderungen zu indizieren, wurde die Summe dieser Faktoren zu T1 von der Summe dieser Faktoren zu T2 subtrahiert, so dass (hohe) positive Werte einen (starken) Anstieg und (hohe) negative Werte eine (starke) Abnahme der Anzahl der Belastungsfaktoren anzeigen. Darüber hinaus wurde geprüft, inwieweit diese Faktoren Veränderungen der negativen Emotionalität der Kinder prädizieren. Hierfür wurden die kontinuierlichen Skalenwerte negativer Emotionalität herangezogen und der Wert zu T1 von dem Wert zu T2 abgezogen.

Für die Analysen wurden nur Fälle mit vollständigen Daten („complete cases“) einbezogen, wobei die Ausfallraten bei den betrachteten Variablen jeweils weit unter 10 % lagen.

## Ergebnisse

### Belastungsprofile und negative Emotionalität der Kinder in der Gesamtstichprobe (T1)

Tab. 1 bietet einen deskriptiven Überblick über die Vergleichsgruppen anhand der einbezogenen Kriterienmerkmale sowie weiterer Belastungsfaktoren. Die Verteilung der so bestimmten Vergleichsgruppen entspricht in hohem Maße der Verteilung, die Lorenz et al. [55] anhand der latenten Klassenanalyse gewonnen hatten. Knapp 2 Drittel der Familien sind als unbelastet eingestuft, rund 12 % sind primär sozioökonomisch belastet (darunter 70 % mit Leistungsbezug nach SGB II), weitere 18 % berichten vor allem Stress und Belastungen in der Elternrolle (darunter 85 % mit Zweifel an erzieherischer Kompetenz) und rund 6 % weisen erhöhte Anteile in allen Risikofaktoren auf (mehrfach belastet). Die durchschnittliche Zahl dieser 14 Kriterienmerkmale variiert in den Gruppen zwischen 0,45 (unbelastete Familien) und 5,86 (mehrfach belastete Familien).

**Table 1:** 

	(1) Unbelastet	(2) Sozioökonomisch belastet	(3) Elternstress- und konfliktbelastet	(4) Mehrfach belastet
Anteil an Gesamtstichprobe	64,8 %	11,9 %	17,6 %	5,8 %
Durchschnittliche Anzahl an Kriteriums-Belastungsfaktoren	0,49	2,93	2,98	5,86
*Sozioökonomische Faktoren*				
Armutsgefährdung (SGB-II-Bezug)	3,79 %	70,16 %	5,32 %	78,11 %
Niedrige Bildung	0,67 %	24,56 %	0,41 %	25,28 %
Beengte Wohnverhältnisse	4,10 %	43,53 %	3,54 %	29,47 %
Alleinerziehend/neuer Partner	1,69 %	32,00 %	1,14 %	41,19 %
Junges Alter der Mutter	1,25 %	27,71 %	0,87 %	34,80 %
Ungeplante Schwangerschaft	9,12 %	59,00 %	11,06 %	67,21 %
Mehrere Kleinkinder im Haushalt	0,30 %	5,36 %	0,63 %	3,70 %
*Konflikte/Elternstress*				
Partnerschaftskonflikte/‑gewalt	2,83 %	8,20 %	25,18 %	38,27 %
Innere Wut/Ärger	2,52 %	1,53 %	42,25 %	53,29 %
Anzeichen Depression/Angststörung	0,20 %	1,57 %	13,07 %	21,66 %
Zweifel an erzieherischer Kompetenz	12,52 %	9,29 %	85,19 %	79,92 %
Negative Attribution gegenüber Kind	2,85 %	5,43 %	24,05 %	43,19 %
Schwierigkeiten, sich in das Kind einzufühlen	4,46 %	2,68 %	50,37 %	45,57 %
Tendenz zur Überreaktion	2,38 %	2,11 %	34,87 %	24,24 %
*Weitere (Belastungs‑)Faktoren*				
Migrationshintergrund	23,37 %	34,02 %	26,44 %	40,28 %
Fehlende soziale Unterstützung	5,21	10,32 %	10,84 %	18,50 %
Negative Kindheitserfahrungen der Bezugsperson	6,46 %	17,39 %	12,90 %	36,42 %
Anzeige eines Elternteils wg. Gewaltdelikt	0,92 %	5,37 %	1,70 %	11,23 %
Hinweise Substanzmissbrauch	0,87 %	5,19 %	2,56 %	10,82 %
Perinatale Probleme	9,93 %	12,34 %	10,01 %	14,59 %
Problematisches Schreiverhalten	0,96 %	2,39 %	3,79 %	8,09 %
Behinderung/Erkrankung	1,11 %	1,39 %	2,13 %	2,19 %
Körperliche Misshandlung	0,71 %	0,34 %	6,63 %	7,75 %
Vernachlässigung	0,24 %	0,50 %	0,73 %	5,88 %
*Durchschnittliche Anzahl aller (Belastungs‑)Faktoren*^*a*^	*0,98*	*3,81*	*3,72*	*7,39*

*SGB-II-Bezug* Bezug von Leistungen zur Sicherung des Lebensunterhalts nach dem Zweiten Buch Sozialgesetzbuch (SGB II). Daten: KiD 0–3 Welle 1 (2015), gewichtete Daten

^a^Unter Einbezug aller hier berücksichtigen Belastungsfaktoren, also nicht nur der Kriteriums-Merkmale für die Gruppenzuordnung

Auch die weiteren Belastungsmerkmale sind am häufigsten in der Gruppe der mehrfach belasteten Familien zu finden. Negative Kindheitserfahrungen der Bezugsperson weisen in dieser Gruppe die höchste Prävalenz auf (36,42 %) und erreichen damit das 6fache des Vergleichswerts unbelasteter Familien. Besonders stark ausgeprägt sind die Unterschiede zwischen diesen beiden Gruppen bei Anzeigen gegen die Eltern wegen Gewaltdelikts, Hinweisen auf Substanzmissbrauch sowie Misshandlung oder Vernachlässigung des Kindes. Die dazugehörigen Werte liegen bei mehrfach belasteten Familien um das 10- bis 20fache über dem Wert für unbelastete Familien. Die beiden anderen Belastungsgruppen nehmen mittlere Positionen ein, wobei Gewaltdelikte und Hinweise auf Substanzmissbrauch etwas seltener und körperliche Misshandlung des Kindes etwas häufiger von elternstressbelasteten Eltern berichtet werden. Die durchschnittliche Gesamtzahl aller in Tab. 1 berichteten Belastungsfaktoren fällt unter mehrfach belasteten Familien mit 7,39 doppelt so hoch aus wie bei sozioökonomisch oder elternstressbelasteten Familien (jeweils rund 3,8 bzw. 3,7), während unbelastete Eltern durchschnittlich nur knapp einen Belastungsfaktor angeben (0,98).

Zu T1 findet sich in mehrfach belasteten Familien der höchste Anteil von Kindern mit hoher negativer Emotionalität (18,28 %), aber auch bei elternstressbelasteten Familien liegt deren Anteil mit 12,00 % deutlich über dem Wert für primär sozioökonomisch belastete Familien (1,82 %), die sich ihrerseits in der negativen Emotionalität der Kinder nicht von unbelasteten Familien (1,49 %) unterscheiden. Diese Unterschiede zwischen elternstress- und sozioökonomisch belasteten Familien sind bemerkenswert, da die durchschnittliche Anzahl der Belastungsfaktoren in diesen beiden Gruppen gleich hoch ausfällt (Tab. 1).

Tab. 2 dokumentiert die 4 logistischen Regressionen zu Prädiktoren hoher negativer Emotionalität der Kinder. Durchgängig weisen Kinder in elternstress- und mehrfach belasteten Familien ein mindestens rund 7fach erhöhtes Risiko hoher negativer Emotionalität verglichen mit Kindern aus unbelasteten Familien auf, während der Unterschied zwischen unbelasteten und primär sozioökonomisch belasteten Familien statistisch unbedeutend ist.

**Table 2:** 

Indikator	Modell 1		Modell 2		Modell 3		Modell 4	
	OR	*p*	OR	*p*	OR	*p*	OR	*p*
Unbelastet (Ref.)	–	–	–	–	–	–	–	–
Sozioökonomisch belastet	1,21	0,654	1,19	0,668	1,04	0,927	0,95	0,893
Elternstressbelastet	8,31	< 0,001	8,38	< 0,001	7,48	< 0,001	6,95	< 0,001
Mehrfach belastet	12,87	< 0,001	13,00	< 0,001	8,61	< 0,001	6,81	< 0,001
Alter Kind	1,03	< 0,001	1,03	< 0,001	1,04	< 0,001	1,04	< 0,001
Geschlecht Kind: weiblich	0,92	0,585	0,93	0,621	0,88	0,425	0,90	0,521
Migrationshintergrund	1,40	0,038	1,40	0,037	1,23	0,243	1,22	0,279
Perinatale Probleme	–	–	1,52	0,081	1,42	0,202	1,37	0,257
Behinderung/Erkrankung Kind	–	–	1,60	0,311	0,79	0,647	0,81	0,671
Problematisches Schreiverhalten	–	–	–	–	27,52	< 0,001	28,39	< 0,001
Unglückliche Kindheitserfahrungen Bezugsperson	–	–	–	–	–	–	2,05	0,002
*Modellmerkmale*								
*N*	5326		5281		5264		5246	
F‑Test	34.912***		29.145***		41.096***		37.964***	
R^2^	0,235		0,241		0,285		0,286	

Datenbasis: KiD 0–3, Welle 1 (T1), gewichtete Daten mit einer Designgewichtung, um Unterschiede in den Bundesländern zu adjustieren, und einer Poststratifizierungsgewichtung (Alter, Staatsangehörigkeit, Schulbildung, Berufsbildung der Mutter, Haushaltskonstellation)

*N* Stichprobenumfang, *OR* Odds Ratios, *R*^*2*^ aufgeklärte Varianz

Signifikanz: *** *p* < 0,001, ** *p* < 0,01, *p < 0,05, ^+^*p* < 0,10

^a^Dichotomisierter Indikator (mindestens 15 Punkte auf einer Skala 6–23)

Auch ein höheres Alter des Kindes geht mit einem erhöhten Risiko negativer Emotionalität einher. Der in Modell 1 und 2 signifikante Effekt des Migrationshintergrundes verliert sich, sobald in Modell 3 problematisches Schreiverhalten der Kinder als Prädiktor einbezogen wird. Dieser Aspekt von kindlichen Regulationsproblemen ist eng mit einer hohen negativen Emotionalität der Kinder verbunden und erklärt einen Teil deren höherer Prävalenz in mehrfach belasteten Familien (Reduktion der Odds Ratio von 13,00 in Modell 2 auf 8,61 in Modell 3). Unglückliche Kindheitserfahrungen der Bezugsperson erweisen sich als ein zusätzlicher Risikofaktor für negative Emotionalität der Kinder, wobei die Ergebnisse für Modell 3 erhalten bleiben. Insgesamt erklären die Modelle 3 und 4 mehr als ein Viertel der Varianz.

### Belastungslagen und negative Emotionalität der Kinder im Längsschnitt

#### *Prädiktoren hoher negativer Emotionalität zu T2*.

Eine hohe negative Emotionalität zu T1 erweist sich als der stärkste Prädiktor für hohe negative Emotionalität zu T2 (Tab. 3). Darüber hinaus steigt das Risiko hoher negativer Emotionalität mit jedem zusätzlichen Belastungsfaktor zu T1 um 50 % an (OR = 1,48). Auch die Dynamik der Belastungsfaktoren ist in vergleichbarem Ausmaß relevant (OR = 1,67), d. h., die Wahrscheinlichkeit hoher negativer Emotionalität der Kinder steigt mit einer Zunahme von Belastungsfaktoren zwischen T1 und T2.

**Table 3:** 

	(1) Logistische Regression hohe negative Emotionalität T2		(2) Lineare Regression Veränderung negativer Emotionalität T2-T1	
	OR	*p*	β	*p*
Negative Emotionalität T1	5,42	0,005	*−0,27*	< 0,001
Alter Kind	0,97	0,086	*−0,25*	< 0,001
Geschlecht Kind (weiblich)	0,69	0,293	−0,01	0,749
Migrationshintergrund	1,01	0,977	−0,01	0,802
Kumulierte Belastungen T1	1,48	< 0,001	0,12	0,002
Veränderung Belastungen T2-T1	1,67	0,001	0,24	< 0,001
F‑Test	29,74***		28,47***	
R^2^	0,103		0,202	

Datenbasis: KiD 0–3, Welle 1 (T1), gewichtete Daten mit einer Designgewichtung, um Unterschiede in den Bundesländern zu adjustieren, und einer Poststratifizierungsgewichtung (Alter, Staatsangehörigkeit, Schulbildung, Berufsbildung der Mutter, Haushaltskonstellation);

Anmerkung: In Analyse 1 geht für negative Emotionalität als abhängige und unabhängige Variable der dichotome Indikator ein, in Analyse 2 wird der kontinuierliche Indikator verwendet

*N* Stichprobenumfang, *OR* Odds Ratio, *β* standardisierter Regressionskoeffizient, *R*^*2*^ aufgeklärte Varianz

Signifikanz: *** *p* < 0,001, ** *p* < 0,01, *p < 0,05, ^+^*p* < 0,10

#### Prädiktoren zunehmender negativer Emotionalität zwischen T1 und T2.

Ein Anstieg (kontinuierlich erfasster) negativer Emotionalität zwischen T1 und T2 tritt weniger bei hohem Ausgangswert zu T1 und weniger bei älteren Kindern auf, die in den querschnittlichen Analysen (Tab. 2) häufiger eine hohe negative Emotionalität aufweisen. Zusätzlich zeigt sich vor allem bei einem Anstieg der Belastungsfaktoren auch ein Anstieg kindlicher negativer Emotionalität. Auch bei einem höheren Ausgangsniveau kumulierter Belastungen zu T1 nimmt die negative Emotionalität der Kinder signifikant stärker zu.

## Diskussion

Die hier vorgestellten Analysen der Studie KiD 0–3 knüpfen an die umfangreiche Forschung zu belastenden Kindheitserfahrungen an, die sich vor allem in ihrer Kumulation als deutlicher Risikofaktor für Beeinträchtigungen der körperlichen und psychischen Gesundheit sowie der Entwicklungs- und Bildungschancen von Kindern erwiesen haben. Entsprechende Erkenntnisse zu Säuglingen und Kleinkindern fehlten jedoch bislang. Mit Fokus auf eine hohe negative Emotionalität von Säuglingen und Kleinkindern betrachten wir frühe Anzeichen psychischer Beeinträchtigungen, die sich auch für spätere psychische Gesundheit im Kindes- und Jugendalter als prädiktiv erwiesen haben [15].

Erwartungsgemäß zeigen die Befunde, dass sich häufiger eine auffällig hohe negative Emotionalität junger Kinder bei kumulierten familialen Belastungsfaktoren findet. Zudem hat sich auch das inhaltliche Profil der Belastungsfaktoren als relevant erwiesen. Im Einklang mit dem Family Stress Model weisen Kinder in elternstressbelasteten Familien deutlich häufiger eine hohe negative Emotionalität auf als Kinder aus primär sozioökonomisch belasteten Familien, wobei in beiden Gruppen durchschnittlich rund 4 Belastungsfaktoren berichtet wurden. Für Kinder, deren Familien mit beiden Arten von Belastungsfaktoren konfrontiert waren, wurde am häufigsten eine hohe negative Emotionalität berichtet. Gleichzeitig ist festzuhalten, dass selbst unter Mehrfachbelastungen nur eine Minderheit der Kinder diese emotionalen Auffälligkeiten zeigen.

Auch kindbezogene Faktoren, insbesondere exzessives Schreien, sowie negative Kindheitserfahrungen der Bezugsperson gehen mit mehr negativer Emotionalität der Kinder einher. Die Gruppenunterschiede haben jedoch auch unter Kontrolle dieser Faktoren Bestand, erweisen sich also als robust. Entsprechend scheint problematisches Schreiverhalten der Kinder nicht der zentrale Faktor zu sein, der Problemen der Eltern in der Elternrolle zugrunde liegt und die emotionalen Belastungen der Kinder in diesen Familien erklärt. Auch unglückliche Kindheitserfahrungen der Bezugsperson erweisen sich als ein zusätzlicher Risikofaktor für negative Emotionalität der Kinder und deuten damit auf die intergenerationale Nachwirkung elterlicher Kindheitserfahrungen hin.

Die Querschnittsbefunde werden durch die Längsschnittanalysen gestützt. Diese bestätigen, dass mit einem Anstieg der Belastungsfaktoren auch die negative Emotionalität der Kinder steigt. Ein hohes Ausgangsniveau der Belastungsfaktoren war ebenfalls mit einer stärkeren Zunahme negativer Emotionalität verbunden. Hierbei muss beachtet werden, dass ein stärkerer Ausfall von sozioökonomisch und mehrfach belasteten Familien im Längsschnitt möglicherweise zu einer Unterschätzung der Effekte führen könnte.

Einschränkend ist anzumerken, dass Angaben zur Emotionalität der Kinder von den Eltern gemacht wurden, deren Urteil durch eigene Belastungen gefärbt sein kann. Auch dass alle Informationen aus einer Quelle – dem Bericht eines Elternteils – stammen, könnte zu einer Überschätzung der Effekte führen. Beobachtungsdaten würden größere Klarheit schaffen. Gleichwohl bieten die Daten von KiD 0–3 wertvollen Aufschluss über die Belastungslagen von Familien, deren Bedeutung für die Entwicklung der Kinder und auch die Wirkung Früher Hilfen. So konnten für die längerfristig aufsuchende Begleitung belasteter Familien im Rahmen der Frühen Hilfen längsschnittlich positive Effekte auf die Emotionalität der Kinder aufgezeigt werden [56]. Bedenkt man die hohen individuellen und gesellschaftlichen Kosten von ACE auch in Deutschland [57], so sind diese Investitionen in die Prävention unglücklicher Kindheiten von zentraler gesellschaftlicher Bedeutung.

## Fazit

Vier Typen von familialen Belastungslagen, die in früheren Analysen mittels latenter Klassenanalysen ermittelt worden waren, konnten auf vereinfachtem Weg anschaulich repliziert werden. Hierbei erweist sich nicht nur die Kumulation von Belastungslagen, sondern auch deren inhaltliches Profil als relevant für die emotionale Entwicklung junger Kinder: Bei hoher Mehrfachbelastung der Familien zeigen Kinder die höchste negative Emotionalität, gefolgt von Familien mit hoher Elternstressbelastung, während bei ausschließlich sozioökonomischen Belastungen keine größeren Schwierigkeiten der Kinder mit der Emotionsregulation zu verzeichnen sind als in unbelasteten Familien. Dies bestätigt das Family Stress Model, nach dem der Persönlichkeits- und Beziehungsdynamik der Familie die größere Relevanz für die psychische und speziell emotionale Gesundheit der Kinder zukommt als den rein sozioökonomischen Faktoren. Zukünftige Forschung zu belastenden Kindheitserfahrungen sollte stärker das inhaltliche Profil familialer Belastungslagen in den Blick nehmen. Insgesamt mangelt es bislang noch an großen europäischen Längsschnittstudien zu belastenden Kindheitserfahrungen, aber auch zur Wirksamkeit präventiver Angebote.

## Footnotes

1 Die Gesamtstichprobe einschließlich anderer Befragungsteilnehmer:innen (Großeltern, Pflegeeltern etc.) umfasste 8063 Kinder.

2 Insgesamt nahmen 945 Bezugspersonen an beiden Befragungen teil.
